# BcL-xL Conformational Changes upon Fragment Binding Revealed by NMR

**DOI:** 10.1371/journal.pone.0064400

**Published:** 2013-05-23

**Authors:** Clémentine Aguirre, Tim ten Brink, Olivier Walker, Florence Guillière, Dany Davesne, Isabelle Krimm

**Affiliations:** 1 UMR5280/Université de Lyon/Université Lyon 1, Institut des Sciences Analytiques, Villeurbanne, France; 2 UMR5822/IN2P3/F-69622 Lyon, Université de Lyon, IPNL, Villeurbanne, France; Russian Academy of Sciences, Institute for Biological Instrumentation, Russian Federation

## Abstract

Protein-protein interactions represent difficult but increasingly important targets for the design of therapeutic compounds able to interfere with biological processes. Recently, fragment-based strategies have been proposed as attractive approaches for the elaboration of protein-protein surface inhibitors from fragment-like molecules. One major challenge in targeting protein-protein interactions is related to the structural adaptation of the protein surface upon molecular recognition. Methods capable of identifying subtle conformational changes of proteins upon fragment binding are therefore required at the early steps of the drug design process. In this report we present a fast NMR method able to probe subtle conformational changes upon fragment binding. The approach relies on the comparison of experimental fragment-induced Chemical Shift Perturbation (CSP) of amine protons to CSP simulated for a set of docked fragment poses, considering the ring-current effect from fragment binding. We illustrate the method by the retrospective analysis of the complex between the anti-apoptotic Bcl-xL protein and the fragment 4′-fluoro-[1,1′-biphenyl]-4-carboxylic acid that was previously shown to bind one of the Bcl-xL hot spots. The CSP-based approach shows that the protein undergoes a subtle conformational rearrangement upon interaction, for residues located in helices 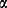
2, 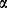
3 and the very beginning of 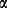
5. Our observations are corroborated by residual dipolar coupling measurements performed on the free and fragment-bound forms of the Bcl-xL protein. These NMR-based results are in total agreement with previous molecular dynamic calculations that evidenced a high flexibility of Bcl-xL around the binding site. Here we show that CSP of protein amine protons are useful and reliable structural probes. Therefore, we propose to use CSP simulation to assess protein conformational changes upon ligand binding in the fragment-based drug design approach.

## Introduction

Protein-Protein Interactions (PPI) play a major role in a large diversity of processes in cells [1]. PPI represent consequently highly attractive targets for the elaboration of chemical probes in chemical biology. PPI are also important therapeutic targets for the design of inhibitors capable of preventing the formation of protein-protein complexes and interfering with biological pathways. However, tackling PPI remains a particularly challenging task in drug design due to the properties of PPI surfaces, by comparison with more typical binding sites of proteins. Protein-protein interfaces happen to be rather flat and large and are therefore less prone to interact with ligands than smaller and deeper pockets found in binding sites of proteins such as enzymes [2]–[6]. A novel approach in drug design called Fragment-Based Drug Design (FBDD) seems to be a very promising methodology and could help developing PPI inhibitors [2], [7], [8]. FBDD consists of screening fragment-like molecules against protein targets, using biophysical methods such as Surface Plasmon Resonance, Nuclear Magnetic Resonance and X-ray crystallography [9], [10]. Fragments are small, simple and very low molecular weight compounds (MW
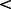
300 Da) that usually bind proteins with low affinity (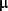
M
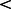
K
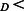
mM). Fragments nevertheless bind proteins through high-quality interactions and display high ligand efficiencies [11], [12]. Potent compounds with improved activities (K
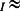
nM) are derived from fragment hits by growing, merging or linking methods [9], [13]. PPI inhibitors resulting from fragment-based approaches have been reported for the Bcl-2 family [14]–[18], for interleukins [19], and for the ZipA/FtsZ interaction [20]. Very recently, FBDD methods have been successfully applied to target the Ras/SOS complex [21], [22] and the BRCA2/RAD51 complex [3].

Protein conformational changes upon ligand interaction make rational drug design even more complicated and challenging. Regarding fragment-like molecules, it is not fully accepted in the scientific community that such ligands can induce protein rearrangement, mostly because they bind proteins with very weak affinities [2]. However, as recently reviewed, resolution of 3D structures of fragment-protein complexes revealed that fragments could induce conformational change, even if they bind proteins with low affinity [23]. All these subtle protein conformational changes upon fragment binding were evidenced by X-ray crystallography, through the comparison of the free protein and the complex structures [2], [23]–[26]. X-Ray is clearly the method of choice for resolving structures, but sometimes it can be difficult to get crystals for protein-fragment complexes. Such structures can also be determined by NMR, using NOESY experiments, but the analysis is much longer and requires the full protein spectrum assignment. Here, we propose to use a very sensitive NMR parameter, the chemical shift, to compare the free and fragment-bound conformations of the protein. The analysis focuses on protein amine groups that can be rapidly assigned. Upon ligand recognition, proton chemical shifts of the protein are perturbed by the change in chemical environment due both to the presence of the ligand and to possible structural changes. The method described in this report relies on the fact that the large majority of fragments contain aromatic moieties. Aromatic rings are responsible for the so-called ring current shift effect [27], which constitutes the major contribution of the ligand-induced proton Chemical Shift Perturbations (CSP) in protein NMR spectra. The ring current shift caused by aromatic rings can be simulated by semi-classical equations, using the Haigh-Maillon model [27]–[30]. Thus, disagreement between experimental fragment-induced CSP and simulated fragment-induced CSP should highlight structural rearrangements of the protein upon interaction. Using 2D ^1^H-^15^N protein spectra, protein residues located in regions that undergo conformational change upon fragment binding will be identified. To illustrate the method, we analyse the interaction of the Bcl-xL protein with a fragment that lead to the discovery of inhibitors ABT737 and ABT263, the most advanced fragment-based application of a PPI inhibitor [16], [31], [32].

Bcl-xL, as a member of the Bcl-2 family of proteins, is involved in the regulation of apoptosis. In normal cells, the anti-apoptotic proteins including Bcl-xL promote cell survival, while the pro-apoptotic members such as Bak and Bad promote cell death [33]–[35]. 3D structures of Bcl-xL bound to peptides from pro-apoptotic proteins showed that the protein undergoes rearrangement upon interaction, involving in particular the shift of the protein helix 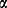
3 [36]–[38]. Upon binding to PPI inhibitors, Bcl-xL structure is also modified, but the conformational change observed on helix 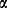
3 depends on the inhibitor size [16], [39], [40]. Regarding fragment-like ligands, Bcl-xL conformational change upon interaction has not been clearly investigated. Fragment-based NMR screening of 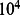 compounds performed against Bcl-xL identified fragment **1** (4′-fluoro-[1,1′-biphenyl]-4-carboxylic acid) as the best hit with a dissociation constant of 300 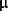
M [15]. The structure of the protein-fragment complex has been calculated by docking the fragment into the protein structure using intermolecular protein-ligand NOEs obtained from ^13^C, ^15^N filtered NOESY experiments (PDB code 1YSG). During the calculation, the protein structure was kept rigid except for binding site residues [15], so the true structure of the protein-fragment complex has not been resolved. More recently, the group of Constantine applied the NOE matching approach to analyse the Bcl-xL/fragment **1** complex [41], [42]. The authors used four different Bcl-xL structures, in the presence of Bak (1BXL), an NMR apo structure (1LXL), a X-Ray apo structure (1MAZ), and the fragment-bound structure 1YSG. The results suggested that the true fragment-bound structure might be more similar to the peptide-bound Bcl-xL structure (1BXL) than the 1YSG structure or the apo structures [42]. Here, to investigate Bcl-xL conformational change upon fragment binding, we compare fragment-induced experimental CSP with simulated CSP resulting from the ring current effect of fragment positions docked in the protein binding site. The analysis reveals that the protein undergoes structural rearrangements involving residues of helices 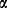
2, 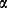
3 and 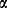
5. To confirm the results and go further into the characterisation of the fragment-induced conformational change, we performed Residual Dipolar Coupling (RDC) experiments, which are sensitive to the orientation of the backbone amine groups [43], [44]. RDC have been previously used to explore protein conformational changes upon protein-protein interactions [45], [46] and protein-ligand interactions [47], [48]. RDCs were measured on the free and fragment-bound forms of Bcl-xL. The comparison of experimental RDCs and back-calculated RDCs on available protein structures confirms subtle Bcl-xL conformational changes upon fragment interaction. Thus, we propose to use proton CSPs as structural probes for investigating protein conformational changes upon fragment binding, and discuss the advantages and limits of the method.

## Results

### Experimental Bcl-xL Chemical Shift Perturbations upon binding to fragment 1

**Figure 1:**
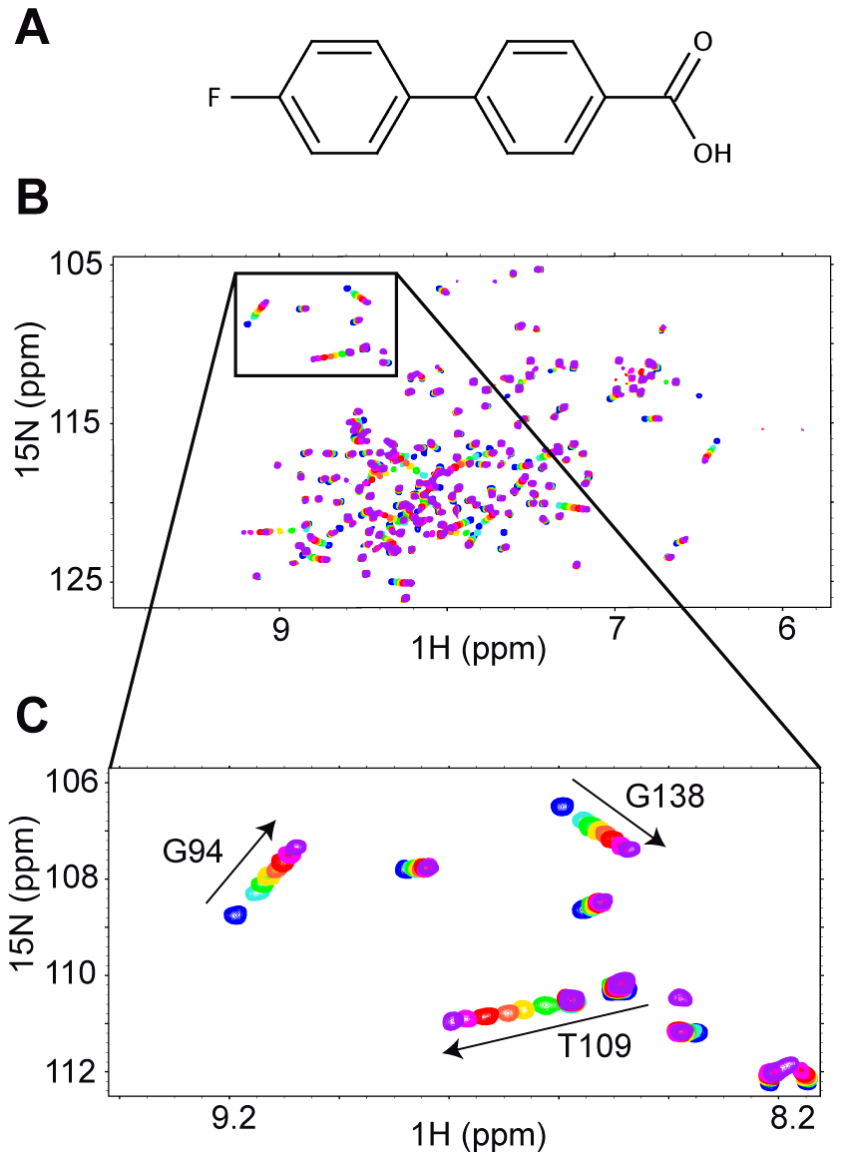
^15^N-HSQC spectra of the Bcl-xL protein in the presence of fragment 1. (A) Structure of fragment **1**. (B) Superimposition of the ^15^N-HSQC protein spectra in the free state (blue) and in the presence of increasing ligand concentration (200 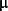
M in cyan, 300 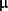
M in green, 500 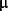
M in yellow, 700 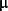
M in orange, 1 mM in red, 2 mM in pink and 4 mM in purple). (C) Section of the ^15^N-HSQC spectrum. Numeration was done according to the PDB code 1R2D.

**Figure 2:**
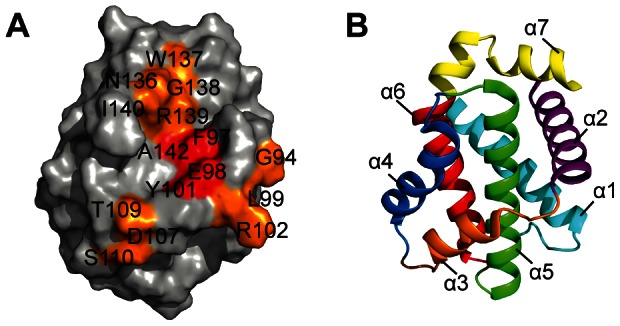
Binding site of fragment 1 mapped onto the protein Bcl-xL surface. (A) The combined shift perturbations CSP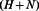 induced by fragment **1** mapped onto the protein surface. Residues that exhibit large perturbations are displayed in red (CSP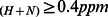) and orange (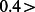
CSP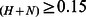 ppm). (B) Ribbon structure of Bcl-xL in the free state (PDB code 1R2D) with the seven helices coloured sequentially.

**Figure 3:**
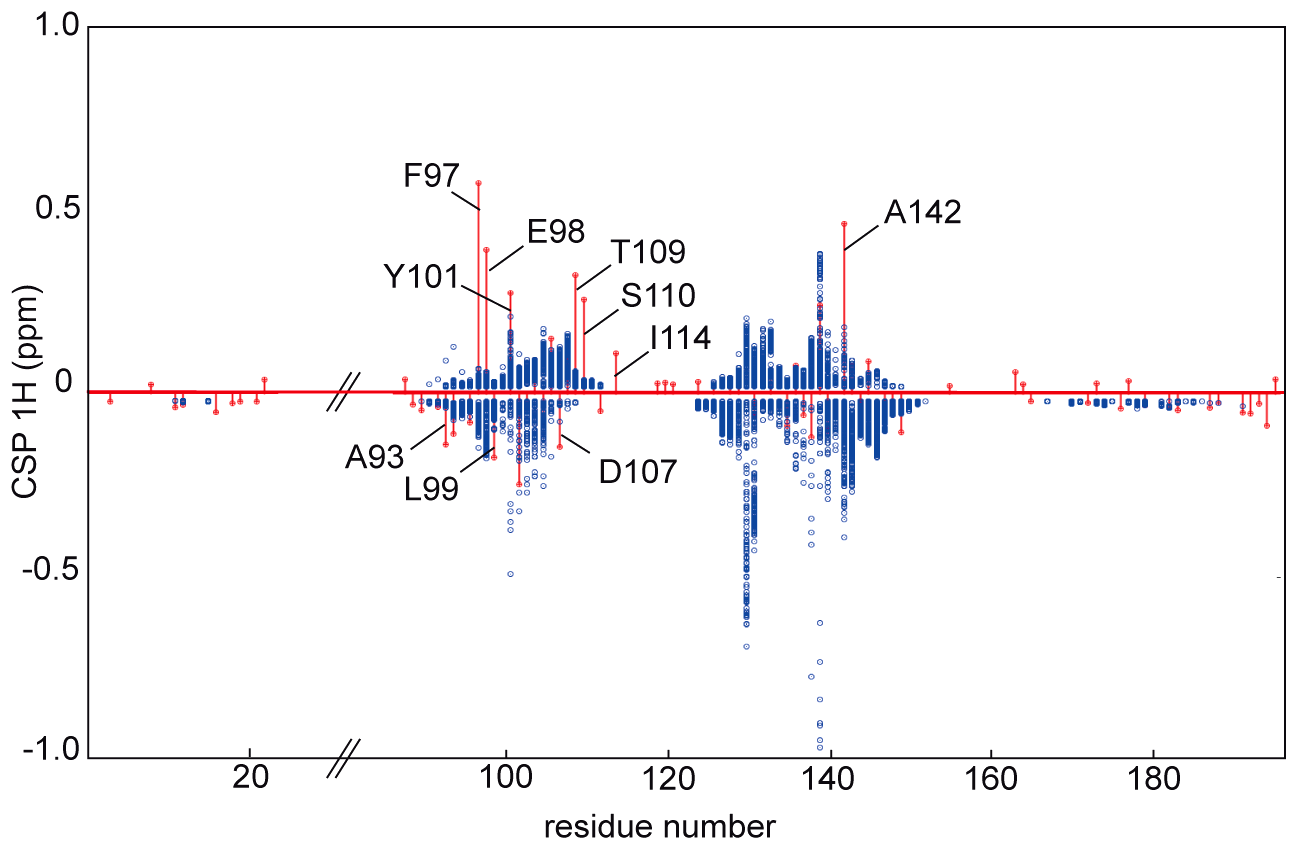
Experimental versus simulated CSP 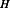 values. Simulated CSP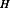 (blue points) are calculated for the 200 structures displayed in Figure 4. Experimental CSP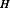 values (red lines) are superimposed to the simulated CSP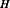 values. Residues 25 to 84 that are absent in the structure 1R2D are removed from the plot.

To investigate Bcl-xL conformational changes upon fragment binding, 2D NMR spectra were recorded for the protein in the absence and the presence of increasing concentrations of the compound **1** (Figure 1). Significant and linear shifts are observed for some of the ^1^H-^15^N cross-peaks, while a large number of residue peaks display no perturbations, indicating a specific binding onto the binding site. Combined CSP including proton and nitrogen resonances (CSP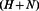) are mapped into the 3D structure of the free Bcl-xL protein in Figure 2 (see equation 3). The binding of fragment **1** induces large chemical shift perturbations for residues located in helices 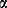
2 (Gly94, Phe97, Glu98, Leu99, Tyr101 and Arg102), 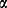
3 (Asp107, Thr109 and Ser110) and 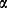
5 (Asn136, Trp137, Glu138, Arg139, Ile140 and Ala142). CSP are specifically observed for residues located into the groove of Bcl-xL that corresponds to the binding site of the apoptotic proteins [36]–[38]. More precisely, the fragment binding site corresponds to the Bcl-xL preferred hot spot of fragments, as previously demonstrated by fragment-based screening [15] and a NMR-based analysis of fragments resulting from deconstruction of Bcl-xL inhibitors [49]. Experimental proton CSP (CSP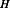) values are displayed in red lines along the protein sequence in Figure 3.

### Generation of Bcl-xL/1 complex structures for the calculation of fragment-induced CSP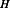 values

**Figure 4:**
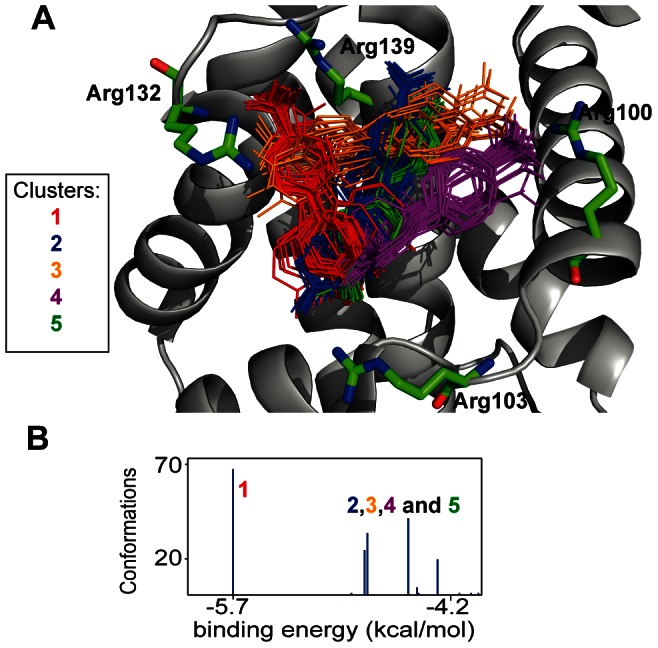
Docked positions of fragment 1 on the protein Bcl-xL binding surface. (A) Superposition of 200 structures of fragment **1** docked into the Bcl-xL binding site. Five clusters are observed (72 poses in red, 37 in blue, 24 in green, 24 in orange and 43 in magenta). (B) Energy binding (in kcal/mol) histogram for the 5 clusters.

To compare experimental CSP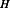 values with fragment-induced simulated CSP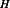 values, 3D structures of Bcl-xL-fragment complexes are required. In the method we use here, the structure of the free protein should be available. To allow a large conformational sampling for the CSP simulation, 200 positions of fragment **1** were generated into the free structure of Bcl-xL binding site (1R2D) using AutoDock software [50]. As illustrated in Figure 4, five clusters are obtained with similar binding energies ranging from −4.3 kcal.mol^−1^ to −5.7 kcal.mol^−1^. Clearly, the binding mode of fragment **1** on Bcl-xL surface is driven by the interaction of the fragment carboxylate with the guanidinium of Bcl-xL arginine residues. Seventy-two out of the 200 positions interact with Arg132 (cluster **1**, red), 37 with Arg139 (cluster **2**, blue), 34 (cluster **3**, orange) and 43 (cluster **4**, purple) with Arg100, while 24 positions interact with Arg103 (cluster **5**, green). In fact, the docked positions in cluster **2** are very similar to the previously NOE-guided docked position of the fragment in the Bcl-xL/**1** complex, as illustrated in Figure S1. In the published model (1YSG), fragment **1** interacts with Bcl-xL through an interaction with Arg139, which correlates with the observation that the modification of the carboxyl group position resulted in a dramatic decrease of affinity [15]. The hydrophobic biphenyl moiety is in contact with residues Phe97, Tyr101, Ala104, Phe105, Leu130, Gly138, Arg139 and Ala142 (Figure S1).

### Comparison of Experimental and Simulated CSP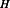 of Bcl-xL upon binding to fragment 1

CSP simulation for the 200 docked structures is shown in blue points in Figure 3. For each amide proton of Bcl-xL, the CSP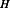 value is simulated for the 200 ligand positions and compared to the experimental CSP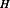 observed for the same Bcl-xL amide proton. In addition, in supplementary material, results are displayed for the five clusters in distinct graphs (Figure S2). As illustrated on Figure 3, significant differences between experimental CSP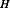 values (red) and CSP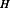 values simulated for the 200 fragment positions (blue points) are observed.

In the approach we report here, we compare experimental CSP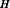 values with simulated CSP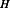 values. Cases where experimental CSP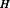 are larger than simulated CSP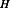 have to be distinguished from cases where the reverse is observed. Simulated CSP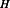 values can become very large when the amine proton is very close (in van der Waals interaction) to an aromatic proton of the ligand (Figure S3). This is the case for example in cluster **1** for residues Leu130 and Phe131 or in cluster **4** for residue Arg139 (Figure S2). Such results are obtained when the docking program places the ligand very close to the protein. Additionally, when saturation is not reached, maximal experimental CSP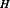 values can be limited by the dissociation constant of the complex, while simulated CSP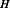 values are calculated for a saturated binding site. Therefore, we focus here on residues for which experimental CSP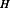 values are larger than simulated CSP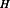 values, as it is unexpected if no protein rearrangement occurs.

As shown in Figure 3, five amide protons located in helix 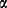
2 (Phe97 and Glu98), helix 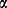
3 (Thr109, Ser110) and helix 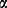
5 (Ala142) exhibit experimental CSP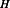 values much larger than the maximal simulated value (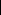
experimental CSP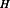 - simulated CSP
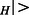
0.2 ppm). In addition, three residues of helix 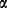
2 (Ala93, Leu99 and Tyr101) as well as two residues of helix 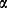
3 (Asp107 and Ile114) exhibit differences larger than 0.06 ppm. When looking separately at each cluster, similar conclusion is drawn. For cluster **1**, cluster **2** and cluster **4**, experimental CSP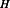 values larger than simulated CSP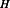 values (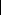
experimental CSP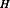 - simulated CSP
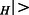
0.2 ppm) are observed for residues 97, 98, 101, 102, 109, 110 and 142. For cluster **3**, residues exhibiting large experimental CSP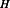 values are 97, 98, 101, 109, 110 and 142, and for cluster **5**, residues are 97, 98, 109, 110 and 142. A P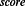 factor was calculated for all the 200 docked positions to estimate the disagreement between experimental and simulated CSP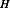 values (see equation 5). The P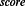 ranges from 0.0069 to 0.0165, and the average P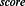 values for the five clusters are similar (0.0087 for cluster **1**, 0.0083 for cluster **2**, 0.0093 for cluster **3**, 0.0082 for cluster **4**, and 0.0092 for cluster **5**), indicating that none of the clusters exhibit a good agreement between experimental and simulated CSP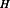 values.

**Figure 5:**
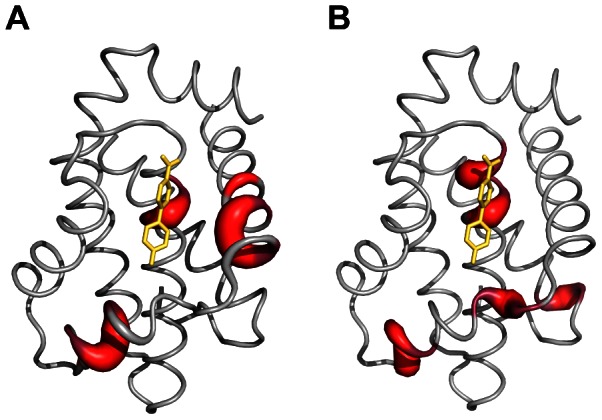
NMR evidences of Bcl-xL conformational change upon fragment binding. (A) CSP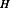 analysis. Significant differences between experimental and simulated CSP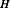 mapped onto the protein surface (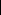
CSP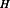-CSP
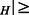
0.06 ppm). (B) RDC analysis. Residues with 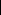
RDC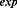-RDC
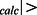
6.5 Hz are displayed.

The residues for which experimental CSP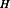 values cannot be explained by the ring current shift of the ligand are mapped on the protein backbone in Figure 5A. The CSP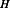 analysis we report here clearly highlights structural rearrangement of Bcl-xL upon fragment binding, likely including backbone movement and side chain reorientation for residues located in helices 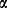
2 and 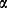
3. In addition, the experimental CSP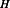 value of residue Ala142 from helix 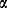
5 does not correlate with any simulated value. Ala142 is located in the Bcl-xL hot spot and very near from the residue Phe97. The latter might slightly move upon interacting with fragment **1**, inducing chemical shift perturbation for Ala142.

### Residual dipolar coupling measurements for the free and fragment-bound forms of Bcl-xL

**Figure 6:**
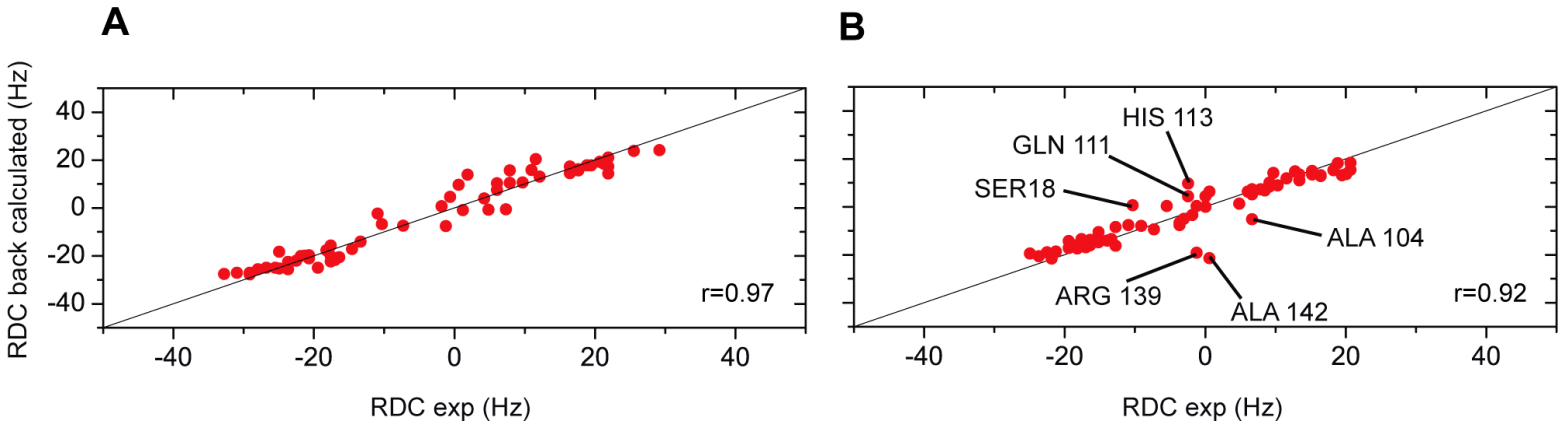
Experimental versus back-calculated RDC on the free protein structure. (A) Comparison of the experimental RDC measured for the free protein and the calculated RDC using the free protein structure 1R2D. (B) Comparison of the experimental RDC measured in the presence of fragment **1** and the back-calculated RDC using the free protein structure 1R2D. Residues exhibiting 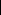
RDC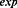-RDC
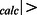
6.5 Hz are labelled.

To confirm the conclusion resulting from the CSP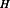 analysis and validate the use of CSP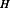 as reliable probes for assessing protein conformational changes upon binding to fragment-like molecules, we also analysed Residual Dipolar Coupling (RDCs) for the free and fragment-bound forms of Bcl-xL. RDCs are a powerful source of long-range orientational information and are very sensitive to structural variations from known protein structures [43]–[45], [47]. The RDC of a ^15^N-^1^1H pair value depends on the orientation of the N-H vectors with respect to the alignment tensor of the molecule and thus the direction of every ^15^N-^1^H bond vector along the protein backbone would provide powerful assessment of the validity of any proposed structure. When 3D structures of proteins are available, RDCs can be back-calculated from the structure and compared with the measured RDCs [45], [47]. Thus, it is possible to compare any form of a protein with a published structure. The correlation coefficient 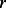 between the experimental RDCs recorded in the absence of fragment and the RDCs back-calculated from the free 1R2D Bcl-xL structure is 0.97 (Figure 6B). This indicates that 1R2D is a good model for the RDC analysis and that the solution structure in our experimental conditions is very similar to the published apo X-Ray structure 1R2D. The correlation slightly decreases to 0.92 when the back-calculated RDCs are compared to RDCs measured in the presence of the fragment. The outliers in the correlation plot correspond to residues of helix 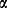
3 (Ala104, Gln111 and His113) and helix 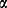
5 (Arg139 and Ala142) (Figure 6). Regarding helix 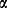
5, all residues but Arg139 and Ala142 exhibit a good correlation between experimental RDCs and back-calculated RDCs, suggesting that helix 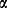
5 does not move. Only the beginning of the helix, which is in direct contact with fragment **1**, is perturbed, in agreement with the CSP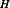 data. Regarding helix 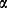
3, three of the residues have a good correlation coefficient, three other residues could not be analysed due to spectral overlapping, and three residues located at both extremities of the helix (Ala104, Gln111 and His113) exhibit significant differences. The RDC measurements therefore confirm that a subtle conformational change upon binding to fragment **1** takes place in the binding region. The NH bonds of the residues with a bad correlation have a different orientation in the bound structure. The RDCs results are therefore in very good agreement with the CSP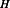 analysis (Figure 5B).

**Figure 7:**
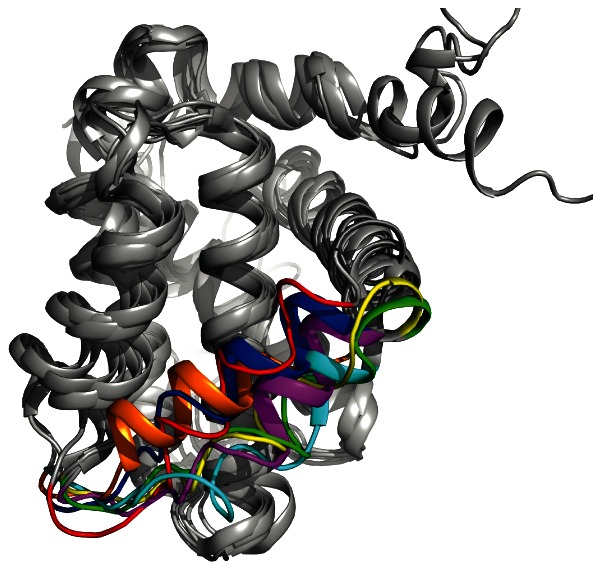
PDB structures of Bcl-xL in free and complexed states. Helix 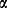
3 that moves upon ligand binding is coloured differently for the ligand-bound structures (1YSG, red; 2O2M, blue and 2YXJ, purple), for the peptide-bound structures (1G5J, cyan; 1BXL, yellow and 2PON, green), and for the free structure (1R2D, orange).

To go further into the analysis of the RDCs, we have focused the analysis on the 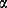
2 and 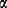
3 helices. Experimental RDCs are now compared to RDCs back-calculated using eight different Bcl-xL structures including apo (PDB codes 1R2D and 1LXL), ligand-bound (PDB codes 1YSG, 2O2M and 2YXJ) and peptide-bound (PDB codes 1G5J, 2PON and 1BXL) structures. The structures are described in Table **1** and compared in Figure 7. The correlation coefficient 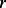 between experimental and back-calculated RDCs ranges from 0.43 for 1G5J to 0.95 for 2O2M, and is 0.64 for 1R2D (Figure 8). The correlation is clearly better with ligand-bound structures (average 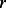 = 0.85) than with the free structures (average 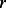 = 0.63) or the peptide-bound structures (average 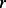 = 0.45). RDC measurements confirm that fragment **1** induces Bcl-xL adaptation upon interaction for residues located in helix 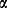
3 and the top of helix 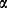
5, and indicate that the rearrangement is more similar to the one observed in the presence of ligands such as in 2O2M than the one observed in the presence of peptide ligands.

**Figure 8:**
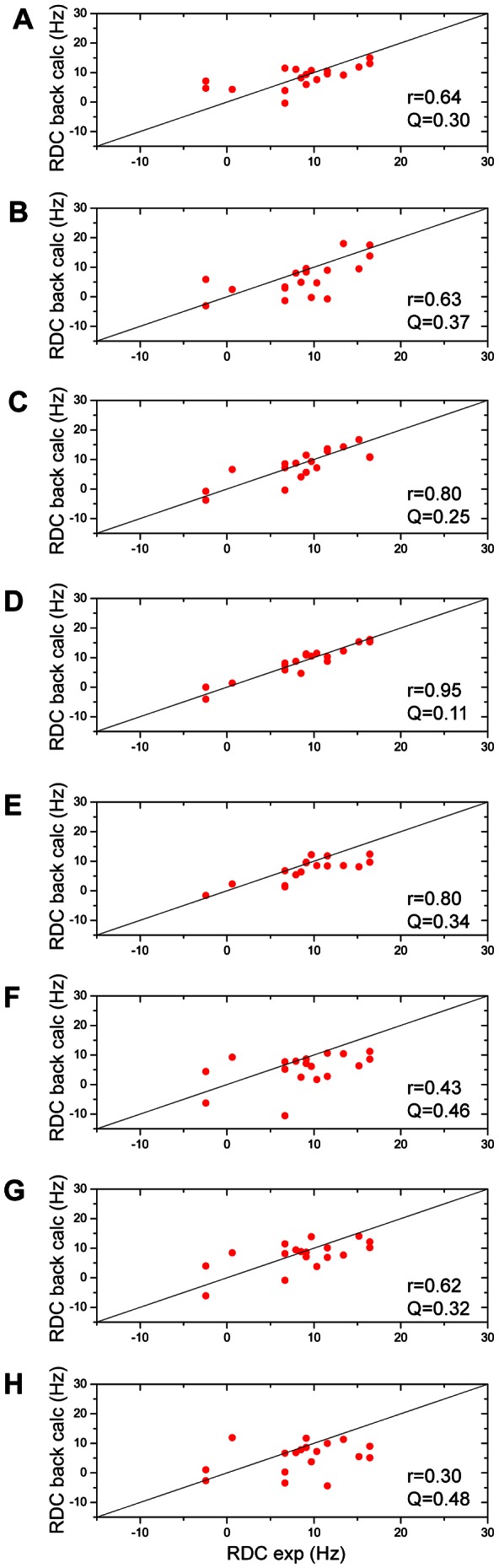
Experimental versus back-calculated RDC for helices 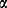
2 and 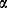
3. Comparison of the experimental RDC measured for the protein-fragment **1** complex and the back-calculated RDC on 8 different PDB structures: (A) 1R2D (B) 1LXL (C) 1YSG (D) 2O2M (E) 2YXJ (F) 1G5J (G) 2PON (H) 1BXL (See Table 1 for more details).

**Table 1:** Protein Bcl-xL available structures.

PDB Code	Structure determination	Ligand	ligand MW (g/mol)	Refs
1R2D	X-ray (resolution 1.95 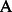 )	none	none	[75]
1LXL	NMR (NOEs+hydrogen bonds+dihedral angle restraints)	none	none	[76]
1YSG	NMR (docking+89 NOEs restraints)	4′-fluoro-1,1′-biphenyl-4-carboxylic acid 5,6,7,8-tetrahydronaphthalen-1-ol	216 148	[15]
2O2M	NMR (docking+94 NOEs restraints)	4-(4-benzyl-4-methoxypiperidin-1-yl)-N-[(4-[1,1-dimethyl-2-(phenylthio)ethyl]amino-3-nitrophenyl)sulfonyl] benzamide	688	[40]
2YXJ	X-ray (resolution 2.2 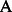 )	4-4-[(4′-chlorobiphenyl-2-yl)methyl]piperazin-1-yl-N-[4-((1R)-3-(dimethylamino)-1-[(phenylthio)methyl]propylamino)-3-nitrophenyl]sulfonylbenzamide	813	[16]
1BXL	NMR (138 NOEs+69 hydrogen bonds+71 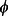 torsional restraints)	BAK peptide (GNVGRNLAIIGDDINR)	1697	[36]
2PON	NMR (NOEs+hydrogen bonds+dihedral angle restraints)	Beclin-1 peptide (GGTMENLSRRLKVTGDLFDIMSG)	2498	[39]
1G5J	NMR (121 NOEs+162 hydrogen bonds+192 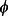 and 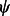 torsional restraints)	BAD peptide (NLWAANRWGRELRRMSDEFVDSFKK)	3113	[37]

For the structure determination by NMR using docking (structures 1YSG and 2O2M), the protein were held fixed with the exception of those residues involved in the binding groove (E96-L112, V127-A142, F191-L194). Peptide sequences are indicated in parenthesis.

## Discussion

Recently, Surade and Blundell reviewed factors that make drug discovery difficult [2]. Among those factors, protein structural adaptation for efficient protein-ligand recognition was considered as a major problem in rational drug design. The authors recall that fragment-like molecules can induce conformation change but this is kinetically and thermodynamically less likely than for drug-like molecules. Protein conformational change can hamper the FBDD approach, since binding sites may be induced by the ligand, or protein conformers may be stabilised from an ensemble upon ligand recognition [2]. To date, few reports deal with fragment-induced protein conformational changes. Using X-ray crystallography, Babaoglu and Shoichet identified fragments that bound very weakly to the AmpC b-lactamase by inducing a novel binding site into the enzyme. The fact that crystallisation conditions were similar to those of the apo structure lead to the suggestion that the observed modification was not an artifact but a specific accommodation of these fragments [25]. Fragment-based screening of the HIV PR also identified fragment binding associated with conformational change in the protein [24]. Recently, a review by Murray and collaborators shows that out of 25 targets, 12 exhibited movement greater than 5 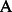 [23]. For example, in HSP90, some fragments induced the so-called collapsed helix formation around residue Gly108. For the well-known case of the BACE protein, fragments can induce significant conformational change in the Glycine-rich loop. Additional protein movement involving Asp168 swinging to form a hydrogen bond with the fragment OH group has also been observed [51]. All these reports were obtained from the resolution of the free and fragment-bound structures of the protein by X-Ray crystallography. Here, we demonstrate using NMR based on CSP and RDC measurements that Bcl-xL undergoes subtle local protein rearrangement upon fragment binding, involving residues of helices 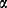
2 and 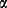
3, and the very beginning of helix 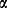
5. This is an additional report showing that fragments can induce protein rearrangements upon interaction.

The RDC measurements show that Bcl-xL conformational change upon fragment binding is not as large as those induced by peptide ligands such as Bak, Bad or Beclin-1 [18], [51], [56] (Figure 8). This is not surprising since helix 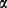
3 shift upon peptide binding was shown to depend on the so-called position P1 of the peptide ligands [39]. This position is not occupied by fragment **1** which binds the protein hot spot [15], [49]. In addition, ligands such as those in structures 2O2M and 2YXJ have different impact on Bcl-xL structures, showing that Bcl-xL clearly adapts its conformation to the ligand [16], [40], [52], [53] (see Table 1 and Figure 7). Here we observe that a small ligand with 16 heavy atoms and a low affinity (K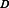 = 300 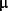
M) induces Bcl-xL movement upon interaction, and this rearrangement is roughly similar to the one observed in a ligand-bound structure (2O2M), at least for the backbone conformation. In general, this raises the question of the intrinsic flexibility of proteins that accommodate various conformations depending on the ligands. For Bcl-xL, the backbone flexibility was recently characterised by MD simulations [52], [53]. Starting from the structures bound to the Bad peptide, the structures did not reach the conformational space shown on the apo structures. Order parameters were compared for apo, holo without the peptide and holo with peptide structures. The region of helices 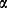
2 and 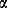
3 is flexible and the backbone flexibility is significantly reduced in the holo structure with peptide; for holo without peptide, higher degrees of flexibility in the loop 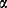
2-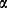
3 and 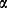
3-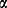
4 are observed when compared to the peptide-bound forms [53]. The higher degree of flexibility around helix 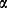
3 revealed in MD simulation correlates with the change of the length and conformation of helix 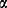
3 when binding to the peptide ligands, and correlates with the fragment-induced conformational changes revealed by our CSP and RDC analysis.

In the absence of protein conformational change, the main contributions to the ligand-induced chemical shift perturbations for proton CSP (CSP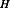) are the ring-current effect of the ligand aromatic rings and the hydrogen bonds between the protein and the ligand [30], [54], [55]. Nitrogen chemical shifts are more difficult to interpret and have not been used yet for analysing protein-ligand complexes. Fragment-like molecules represent simple organic compounds with few chemical functions and a very low molecular weight (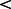
300 Da) that typically contain at least one aromatic ring. The ring current effect will therefore be largely predominant in the fragment-induced proton CSP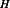. Hydrogen bond effects can be large, but this typically affects few protein residues, while ring-current effects are spread around the aromatic ring up, as illustrated in Figure S3. In addition, simulation of protein-ligand hydrogen bonds is still problematic [55]–[58]. Quantitative analysis of CSP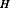 for protein-ligand interactions has been reported for the first time by McCoy and Wyss in 2000 [30]. Ligand-induced CSP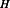 simulation has been used the last decade by three different groups for assessing ligand binding modes [55], [59]–[61]. The CSP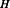 simulation consists of calculating the ring current effect on proton chemical shifts using the Haigh-Mallion model (equation 4). In the published reports, experimental CSP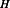 are compared to CSP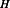 simulated from docked ligand positions with semi-classical methods. The ligand binding mode corresponds to the ligand position exhibiting the best agreement between experimental and simulated CSP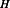 values. Published cases are the complexes formed between calmodulin and a naphthalene derivative using a Trp probe [30], HCV NS3 protease and a series of weak-affinity ligands [59], the protein antitumor antibiotic NSC and a synthetic chromophore analogue [60], barnase and deoxyoligonucleotides [61] and three kinase-ligand complexes [55]. In these examples where the ligand binding mode is deduced from the quantitative analysis of CSP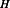, it is required that the protein does not undergo any conformational changes upon ligand binding. If the protein experiences a rearrangement upon interaction, experimental CSP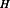 will contain direct contributions from the ligand (ring current shifts) and indirect contributions due to protein conformational rearrangements such as backbone movements and side chain reorientations. For example, when rearrangement involves reorientation of aromatic residues, ring current effects of Phe, Tyr and Trp side chains will contribute to the CSP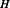 observed. For Bcl-xL, reorientation of aromatic sides chains of residues Phe97, Tyr101 and Phe105 have been observed (Figure S4). The aromatic ring movements could contribute to the large CSP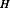 observed for residues in their vicinity. Finally, it is important to notice that RDC measurements on the free protein shows that 1R2D is a good starting structural model; therefore CSP disagreements do not result from divergences between the X-Ray Bcl-xl structure used for the docking and the structure in solution. In addition, RDC measurements complement CSP data as they show that the backbone of Bcl-xL is also modified in the helix 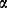
3 upon ligand interaction.

Here we do not solve the structure of the fragment-protein complex but we propose to use a fast NMR method to assess if an induced fit process is observed upon fragment binding. An assigned 2D NMR spectrum is necessary, which is easily feasible for proteins below 50 kDa. In addition, an apo 3D structure of the protein is required. When only a protein structure in a presence of a ligand is available, it is also possible to take the holo structure as the 3D model; disagreements will show that the protein coordinates have changed and that the holo and apo structure differ. For example, CSP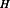 values simulated with the ligand-bound 2O2M Bcl-xL structure clearly highlight similar disagreements between experimental and simulated CSP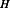 values, with an average P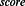 value of 0.008 (Figure S5). Using the apo Bcl-xL structure or the holo 2O2M Bcl-xL structure, experimental CSP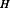 values for residues 97, 98, 101, 109, 110 and Ala142 are never explained by any ligand positions, showing that these residues are located in a region that moves upon ligand recognition. Disagreement between experimental and simulated CSPs larger than 0.2 ppm are observed for these residues. Previously published examples showed that in the absence of conformational change, we should not observe discrepancies between experimental and simulated CSP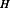 [30], [60], [61].

## Conclusion

Protein-protein interfaces represent therapeutic targets for the future drugs. These interfaces are typically large and flexible, and as a consequence still remain a real challenge for drug discovery. Methods able to detect protein structural rearrangements upon interactions should therefore be useful in this context, to identify proteins subject to conformational change, to locate theses changes, and to compare structural modifications with MD calculations. The results we report here demonstrate that CSP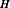 analysis could be routinely used for this purpose.

## Materials and Methods

### Proteins production and purification

The protein Bcl-xL was expressed as a 6His-tagged protein in *Escherichia coli* strain BL21(DE3 gold) using pQE-30 expression vector. *E. Coli* were grown at 37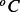 in M9 minimal medium supplemented with thiamine and containing ^15^NH_4_Cl as the sole nitrogen source to produce uniformly ^15^N-labelled protein. The protein expression was induced with isopropyl 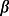-D-1-thiogalactopyranoside (0.5 mM) for 2 h. Cells were then lysed in 20 mM imidazole, 20 mM sodium phosphate, 500 mM NaCl supplemented by lysozyme and DNase (pH = 7.4) by sonification and clarified by centrifugation. The 6His-tagged protein contained in the supernatant was purified onto a His GraviTrap column (GE Healthcare) by Ni^2+^-affinity chromatography. The protein was eluted with 500 mM imidazole, 20 mM sodium phosphate buffer and 500 mM NaCl. The protein buffer was then exchanged by dilution/concentration cycles, against 25 mM sodium phosphate, (pH = 7.0) containing 3 mM DTT (Dithiothreitol).

### CSP measurements

NMR samples contained 200 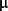
M uniformly ^15^N-labelled Bcl-xL and ligand concentrations from 0 to 4 mM (saturation). The concentration of DMSO-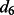 did not exceed 3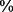 in the NMR tube. 2D ^15^N-HSQC spectra were acquired at 28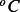 with a Varian Inova 600 MHz NMR spectrometer, equipped with a standard 5 mm triple-resonance inverse probe with z-axis field gradient, using 64 t1 increments. Control 1D spectra preceded all experiments to assess the purity and stability of the fragment. All NMR spectra were processed with the Varian *Vnmrj* and the NMR*Pipe* softwares and analysed using NMR*View* and Sparky [62]–[64].

The proton and nitrogen chemical shift perturbations (CSP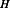 and CSP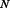, respectively) induced by the fragment were defined as the difference between the chemical shift of the protein in the bound and the free states (see eq.1 and eq.2).
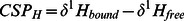
(1)

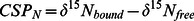
(2)The combined perturbation 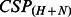 was calculated using the proton (CSP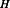) and the nitrogen (CSP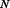) chemical shift perturbations [65]:
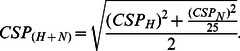
(3)


### Docking calculation

The generation of 200 ligand poses located in the binding site was performed using AutoDock4 with the AutoDockTools4 graphical interface [50]. Grid maps were generated with 0.375 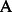 spacing into the protein binding site. The docking calculations were performed using Genetic Algorithm (GA) for ligand conformational searching. The 3D structure used was the apo X-ray structure 1R2D. The protein was kept rigid during the docking procedure.

### CSP simulation

CSP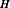 were calculated by means of the Haigh-Maillon semi-classical model [27] (eq.4) widely used for CSP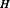 calculation on amide proton and in other popular programs (SHIFTX [28], SHIFTS [29], Shifty [66], CH3Shift [67] and SPARTA [68]):
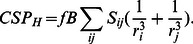
(4)Here, 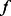 is the ring-specific intensity factor (1.00 for benzene type ring), 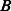 is the target nucleus factor (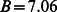 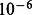 for amide nuclei [28]). Other values for 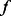 and 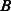 can be found in a recent work [69]. 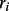 and 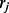 are the distances from the ring atoms 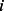 and 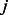 to the proton HN of the protein. 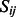 is the area of the triangle formed by atom 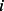 and 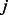 and the HN proton projected onto the plane of the aromatic ring. The sums are over the bonds in the ring.

We wrote a Fortran program to calculate the CSP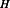 induced by a fragment position on each protein amide proton. The program needs ligand PDB file (provide by docking experiments) and associated protein PDB file to load nucleus coordinates (available formats: .pdb and .pdbqt). To evaluate the agreement of the simulation with experimental CSP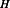 a factor P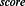 was introduced in the program and calculate for each pose 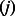:
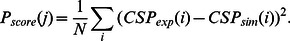
(5)


The number of protein residues was considered by N (N = 196 for Bcl-xL). Low P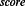 indicates that the docking solution is in good agreement with the experimental CSP. Nevertheless, the ligand-protein complex likely exists as energetically close-states, that are not necessarily discriminated by the CSP and the P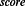 values, especially since simulated CSP only contain ring-current shift contributions.

### RDC

HN RDCs were measured in 25 mM sodium phosphate, 50 mM NaCl, 3 mM DTT at 28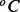 with IPAP-type experiment [70] using 20 mg/mL of Pf1 LP11-92 phage. RDC experiments were performed for the protein Bcl-xL alone (350 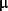
M) and in the presence of the fragment (2 mM). In the presence of anisotropic medium, the ^2^H signal splitting was 20
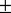
5 Hz for the free and fragment-linked of Bcl-xL. This ensure that similar degree of alignment is used for both. 256 increments t1 were recorded in the indirect dimension ^15^N and RDC values were obtained from the differences in the ^15^N-^1^H coupling observed in the isotropic and anisotropic case. RDCs ranged from −33 Hz to 29 Hz and from −33 Hz to 23 Hz for the protein in the free state and in the presence of the fragment **1** respectively. Residues showing overlapped signals were removed from the analysis that was performed with the in-house program ALTENS [71]. The RDCs were back-calculated as previously published [71], using data collected in both presence and absence of ligand according to the method of Singular Value Decomposition (SVD) [72]. To account for the goodness of the fit, correlation factors 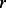 (Pearson factor) and quality factors 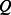 were calculated [73].

## Supporting Information

Figure S1 **Bcl-xL/fragment 1 complex.** (A) Docked Bcl-xL-fragment complex that best mimics the complex structure available in the PDB (included in cluster **2**). (B) Bcl-xL-fragment **1** structure determined by Petros and coworkers (PDB code 1YSG). For both, hydrophobic residues are shown in yellow and residues labelled in bold are involved in hydrogen bond or electrostatic interaction (Ligplot+ analysis [74]).
(TIF)

Figure S2
**Experimental versus simulated CSP** 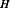 **values.** Experimental CSP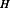 values (red lines) are superimposed to the simulated CSP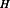 values calculated using the PDB file 1R2D (blue points). Comparison between experimental and calculated results are shown for (A) cluster **1**, (B) cluster **2**, (C) cluster **3**, (D) cluster **4** and, (E) cluster **5**. Residues 25 to 84 are removed from the plot.(TIF)

Figure S3 **Iso-shielding curves of the ring current effect induced by a benzene ring on chemical shift amide proton.** The chemical shift perturbations values calculated according to the Haigh-Mallion theory are labelled on each curve and expressed in ppm. Iso-shielding curves (A) in the benzene ring plane (B) in the plane perpendicular to the benzene ring plane.
(TIF)

Figure S4
**Aromatic rings located in helices** 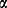 **2 and** 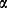 **3 of Bcl-xL.** Each structure is coloured by PDB code to compare the position of (A) Phe97, (B) Tyr101, and (C) Phe105.(TIF)

Figure S5
**Experimental CSP** 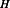 **values versus simulated CSP** 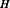 **values calculated with the structure 2O2M.** Simulated CSP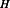 (blue points) are calculated for the 200 structures using the PDB file 2O2M. Experimental CSP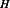 values (red lines) are superimposed to the simulated CSP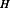 values. Residues 25 to 84 are removed from the plot.(TIF)
