# Supplementary figures and images for: BcL-xL Conformational Changes upon Fragment Binding Revealed by NMR
Source: PLoS One. 2013 May 23;8(5):e64400. doi: 10.1371/journal.pone.0064400 (PMC3662666; doi:10.1371/journal.pone.0064400)

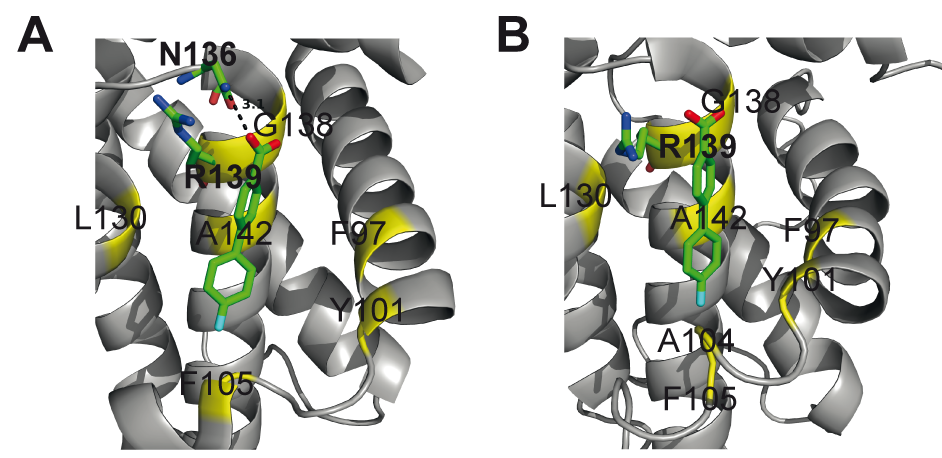

Supplement: Figure S1 — Bcl-xL/fragment 1 complex. (A) Docked Bcl-xL-fragment complex that best mimics the complex structure available in the PDB (included in cluster 2). (B) Bcl-xL-fragment 1 structure determined by Petros and coworkers (PDB code 1YSG). For both, hydrophobic residues are shown in yellow and residues labelled in bold are involved in hydrogen bond or electrostatic interaction (Ligplot+ analysis [74]). (TIF) [file pone.0064400.s001.tif]

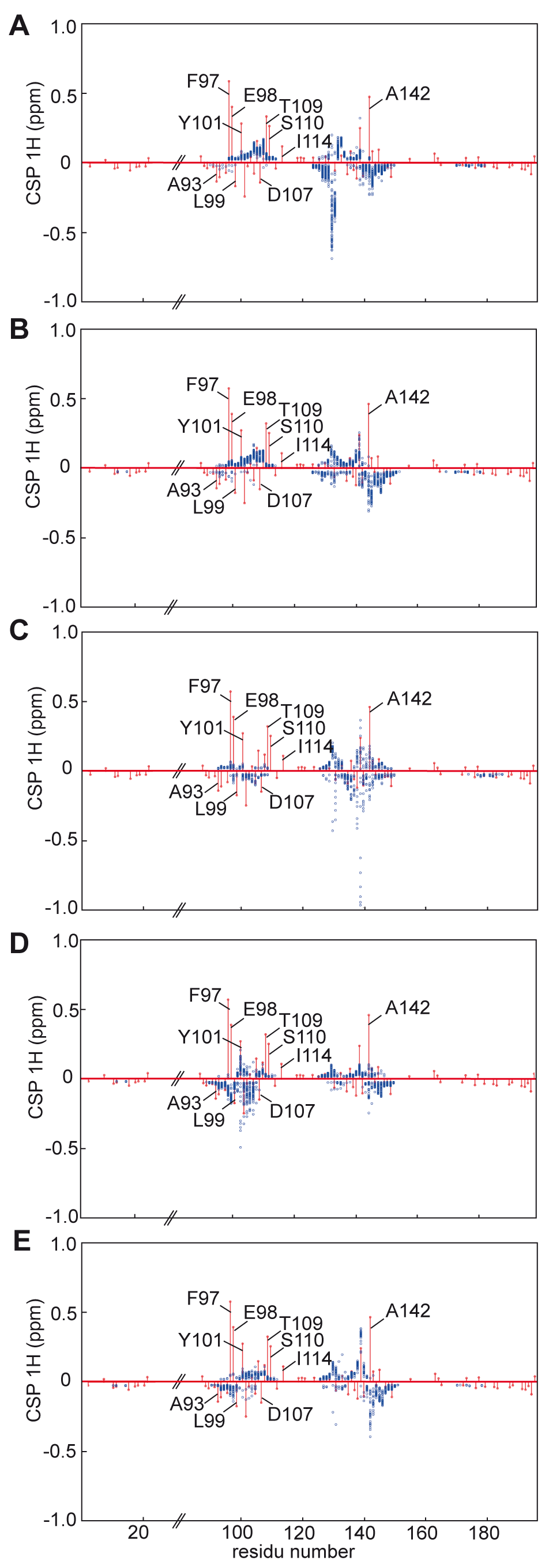

Supplement: Figure S2 — Experimental versus simulated CSP values. Experimental CSP values (red lines) are superimposed to the simulated CSP values calculated using the PDB file 1R2D (blue points). Comparison between experimental and calculated results are shown for (A) cluster 1, (B) cluster 2, (C) cluster 3, (D) cluster 4 and, (E) cluster 5. Residues 25 to 84 are removed from the plot. (TIF) [file pone.0064400.s002.tif]

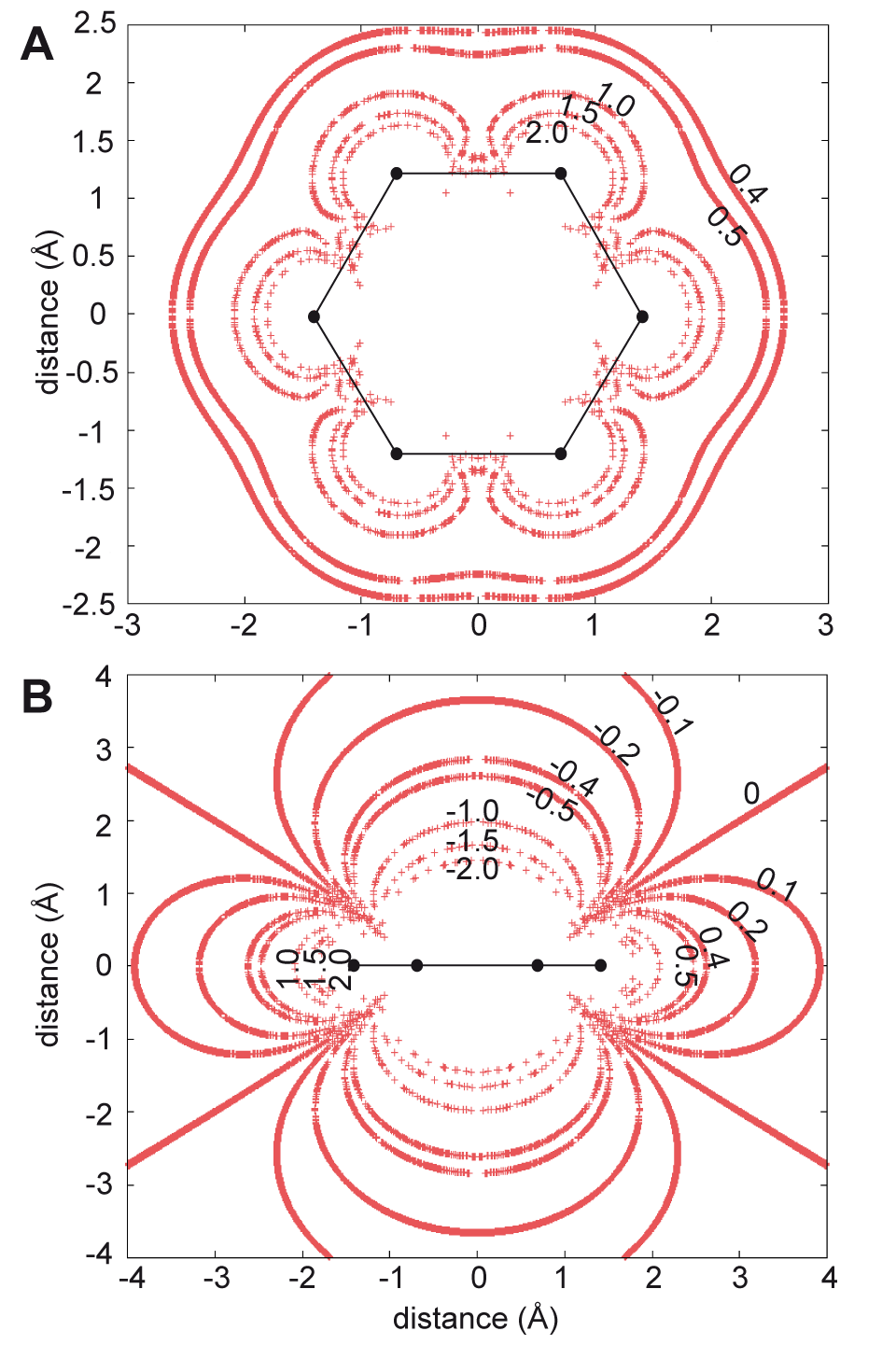

Supplement: Figure S3 — Iso-shielding curves of the ring current effect induced by a benzene ring on chemical shift amide proton. The chemical shift perturbations values calculated according to the Haigh-Mallion theory are labelled on each curve and expressed in ppm. Iso-shielding curves (A) in the benzene ring plane (B) in the plane perpendicular to the benzene ring plane. (TIF) [file pone.0064400.s003.tif]

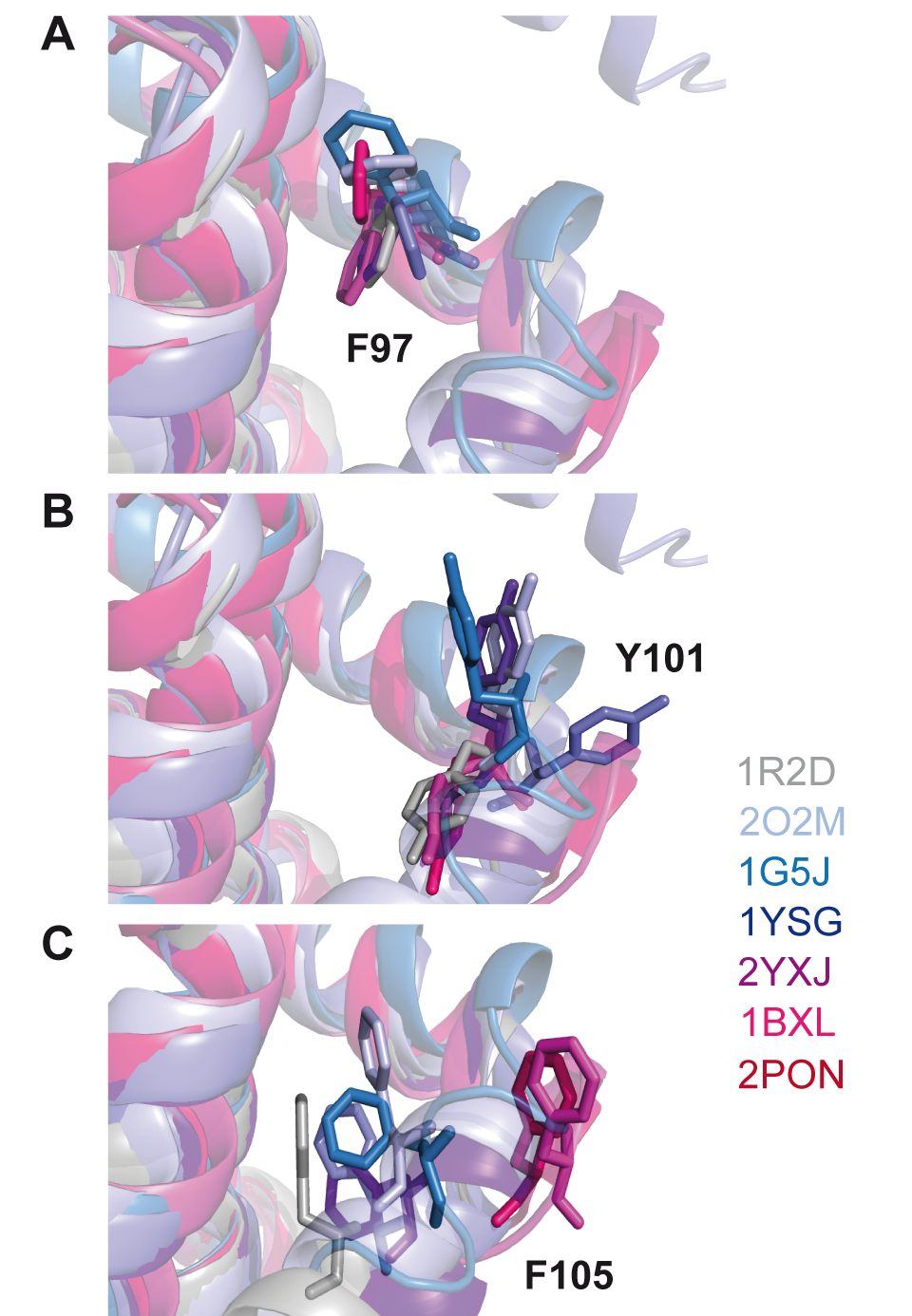

Supplement: Figure S4 — Aromatic rings located in helices 2 and 3 of Bcl-xL. Each structure is coloured by PDB code to compare the position of (A) Phe97, (B) Tyr101, and (C) Phe105. (TIF) [file pone.0064400.s004.tif]

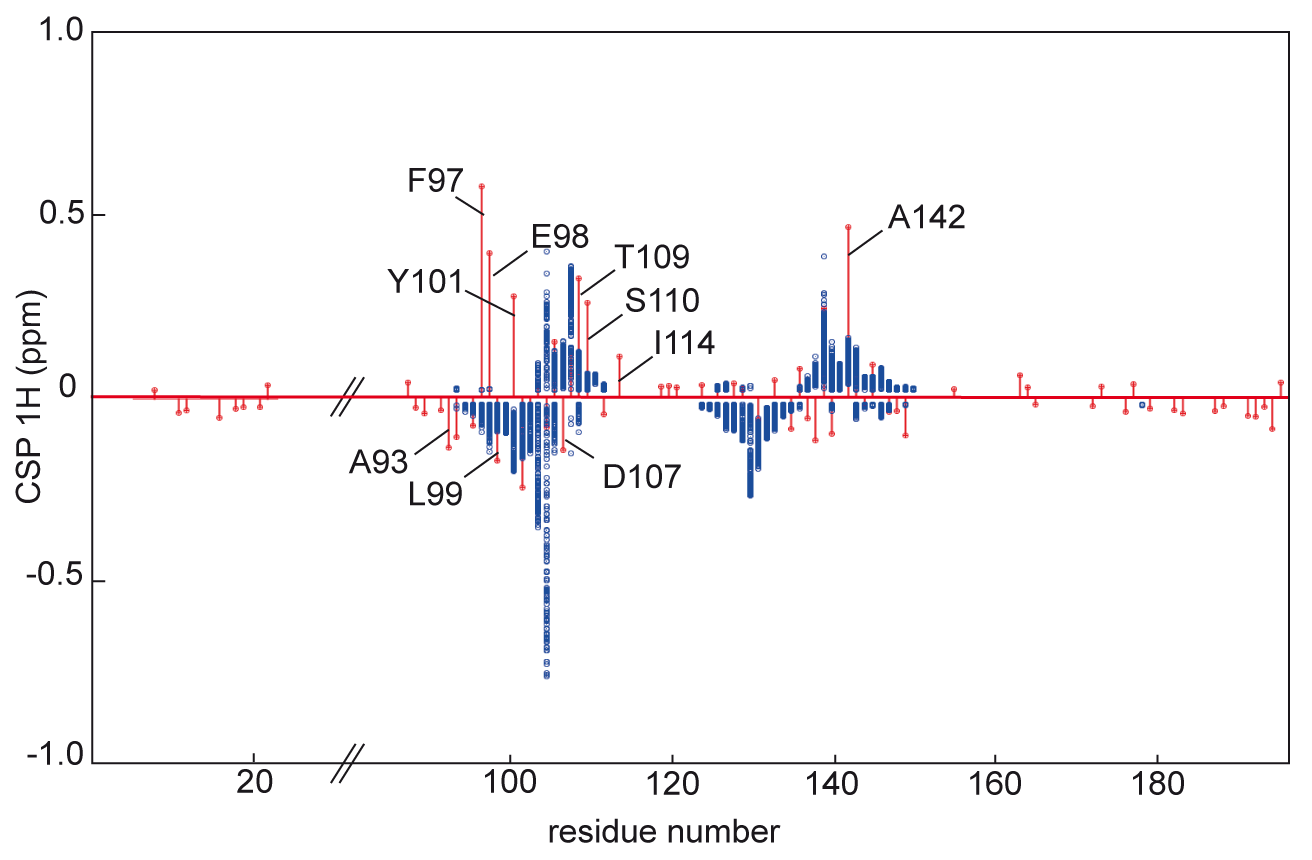

Supplement: Figure S5 — Experimental CSP values versus simulated CSP values calculated with the structure 2O2M. Simulated CSP (blue points) are calculated for the 200 structures using the PDB file 2O2M. Experimental CSP values (red lines) are superimposed to the simulated CSP values. Residues 25 to 84 are removed from the plot. (TIF) [file pone.0064400.s005.tif]
